# Role of Endocannabinoids in Energy-Balance Regulation in Participants in the Postobese State—a PREVIEW Study

**DOI:** 10.1210/clinem/dgaa193

**Published:** 2020-04-25

**Authors:** Mathijs Drummen, Lea Tischmann, Blandine Gatta-Cherifi, Daniela Cota, Isabelle Matias, Anne Raben, Tanja Adam, Margriet Westerterp-Plantenga

**Affiliations:** 1 Department of Nutrition and Movement Sciences, NUTRIM, School of Nutrition and Translational Research in Metabolism, Maastricht University, MD, the Netherlands; 2 Endocrinology Department, Haut-Lévêque Hospital, Pessac, France; 3 INSERM, Neurocentre Magendie, Physiopathologie de la Plasticité Neuronale, Bordeaux, France; 4 University of Bordeaux, Neurocentre Magendie, Physiopathologie de la Plasticité Neuronale, Bordeaux, France; 5 Department of Nutrition, Exercise and Sports, University of Copenhagen, Copenhagen, Denmark

**Keywords:** endocannabinoids, energy balance, adiposity, protein

## Abstract

**Context:**

Endocannabinoids are suggested to play a role in energy balance regulation.

**Objective:**

We aimed to investigate associations of endocannabinoid concentrations during the day with energy balance and adiposity and interactions with 2 diets differing in protein content in participants in the postobese phase with prediabetes.

**Design and Participants:**

Participants (n = 38) were individually fed in energy balance with a medium protein (MP: 15:55:30% of energy from protein:carbohydrate:fat) or high-protein diet (HP: 25:45:30% energy from P:C:F) for 48 hours in a respiration chamber.

**Main Outcome Measures:**

Associations between energy balance, energy expenditure, respiratory quotient, and endocannabinoid concentrations during the day were assessed.

**Results:**

Plasma-concentrations of anandamide (AEA), oleoylethanolamide (OEA), palmitoyethanolamide (PEA), and pregnenolone (PREG) significantly decreased during the day. This decrease was inversely related to body mass index (AEA) or body fat (%) (PEA; OEA). The lowest RQ value, before lunch, was inversely associated with concentrations of AEA and PEA before lunch. Area under the curve (AUC) of concentrations of AEA, 2-AG, PEA, and OEA were positively related to body fat% (*P* < .05).

The HP and MP groups showed no differences in concentrations of AEA, OEA, PEA, and PREG, but the AUC of 2-arachidonoylglycerol (2-AG) was significantly higher in the HP vs the MP group.

**Conclusions:**

In energy balance, only the endocannabinoid 2-AG changed in relation to protein level of the diet, whereas the endocannabinoid AEA and endocannabinoid-related compounds OEA and PEA reflected the gradual energy intake matching energy expenditure during the day.

Weight gain and obesity are known risk factors for type 2 diabetes (T2D). The progression of prediabetes to T2D in individuals with overweight or obesity can be prevented by sustained weight loss (1-3). However, the most effective lifestyle approach to achieve this goal has not yet been clearly defined. Therefore, the main objective of the PREVIEW study (PREVention of diabetes through lifestyle Intervention and population studies in Europe and around the World) (4) was to determine whether a high-protein (HP) low-glycemic index diet was more effective in preventing T2D compared to a moderate-protein (MP), higher-glycemic index diet for weight maintenance. Another objective was to determine whether shorter, but increased intensity of physical activity (PA) had an additional beneficial effect on the outcomes compared with moderate-intensity (MI) PA.

The endocannabinoid system (ECS) has been suggested to play a role in the regulation of energy balance in humans (5). The ECS encompasses the endocannabinoid type receptor 1 and 2 (CBRs), the endocannabinoids, and the pathways responsible for the synthesis and degradation of those ligands (5-7). Endocannabinoids, of which the most studied are arachidonoylethanolamide (anandamide, AEA) and 2-arachidonoylglycerol (2-AG), are polyunsaturated fatty acids produced on demand from membrane phospholipids to act on CBR in an autocrine or paracrine manner (8). CB1R is widely distributed in the brain and also in peripheral tissues such as adipose tissue, liver, the gastrointestinal tract, pancreas, and skeletal muscles (5-12), and is suggested to promote obesity (5). Interestingly, by acting both peripherally and centrally and by affecting the actions of leptin and insulin, CB1R is a potential therapeutic target against obesity and T2D (7-10, 13-17). Plasma AEA is positively associated with adiposity (17), and plasma 2-AG is associated with ghrelin levels (18), whereas plasma oleoylethanolamide (OEA) and palmitoyethanolamide (PEA), 2 endocannabinoid-related compounds, are suggested to act opposite AEA and 2-AG, and may stimulate satiety or energy expenditure (17). Finally, the neurosteroid pregnenolone acts as a signaling-specific inhibitor of CB1R and is part of an endogenous negative feedback loop that decreases the activity of the receptor (19, 20).

The endocannabinoids and their related compounds can be modulated by the diet and in particular by polyunsaturated fatty acid content (5). We recently observed that postprandial 2-AG concentrations were higher after a diet with an increased protein-to-carbohydrate-ratio, whereas AEA, OEA, PEA, and PREG concentrations were not affected by the macronutrient content of the diet (21). However, we did not explore possible associations between the ECS and energy balance or adiposity in this group of participants. Therefore, the aim of the present study was to investigate possible associations of endocannabinoid concentrations during the day with energy balance and adiposity in a subgroup of participants with prediabetes in the postobese phase of the PREVIEW study.

## Materials and Methods

The Medical Ethical Committee of Maastricht University approved the PREVIEW study, registered at clinicaltrials.gov with identifier NCT01777893, as well as the respiration chamber substudy. The study was performed in line with the Declaration of Helsinki. All volunteers signed written informed consent.

### Participants

Forty individuals were recruited from the PREVIEW study population at Maastricht University in the Netherlands, of whom 2 dropped out because of lack of time. For the general PREVIEW study participants underwent a screening that included anthropometric measurements as described in Fogelholm et al (4). Additional exclusion criteria for the respiration chamber study were claustrophobia, smoking, and previous cardiovascular events.

### Study design

The PREVIEW study design was composed of a 3-year, multinational, randomized trial with 4 intervention arms in a 2 × 2 factorial design in 8 intervention centers (Denmark, Finland, United Kingdom, the Netherlands, Spain, Bulgaria, Australia, and New Zealand, with 2326 adults (age 25-70 years, body mass index, BMI ≥ 25 kg/m^2^) with prediabetes as defined by the American Diabetes Association criteria: fasting plasma glucose 5.6 to 6.9 mmol/L and/or 7.8 to 11.0 mmol/L at 2 hours after an oral glucose tolerance test of 75 g glucose, with a fasting plasma glucose concentration less than 7.0 mmol/L (4). A total of 962 participants completed the 3-year intervention starting with a 2-month 8% or more weight reduction phase using a low-energy diet (22) followed by a randomized 34-month weight maintenance phase in 1 of the 4 treatment arms: HP-HI PA, HP-MI PA, MP-HI, and MP-MI; (MP: 15:55:30% of energy from protein:carbohydrate:fat; HP: 25:45:30% of energy from protein:carbohydrate:fat), with the main outcome measures being incidence of T2D over 3 years analyzed by diet and PA treatment, subsequently according to diet and PA, and secondarily among others changes in body weight, BMI, body composition, and insulin resistance (homeostasis model assessment for insulin resistance, HOMA-IR) (4). Close to the last clinical investigation day of the weight maintenance period, approximately 34 months after starting the PREVIEW weight maintenance intervention, participants stayed in the respiration chamber for 48 hours. The 38 participants participating in the respiration chamber measurements had lost on average 11.1 ± 3.6 kg (11.9 ± 2.5%; range, 8.1-18.2%) during the weight loss period. After the subsequent 34-month weight maintenance phase, the average body weight was still 5.5 ± 6.2 kg lower compared to baseline, corresponding with a BMI of 28.9 ± 3.9 kg/m^2^ and an average regain in body weight of 5.6 kg during the weight maintenance phase. There were no differences between the 2 dietary intervention groups, HP and MP, regarding changes in body weight during the PREVIEW intervention (Table 1).

### Respiration chamber

Participants arrived at the Metabolic Research Unit Maastricht research facilities in the morning having fasted overnight from 10 pm the night before. The respiration chamber session started at 9:30 am and stopped 2 days later at 9:30 am. The respiration chamber is an airtight chamber of 14 m^3^ furnished with a bed, chair, desk with computer, television, telephone, intercom, sink, and toilet. The climate inside the chamber was controlled. O_2_ consumption and CO_2_ production were continuously measured by open-circuit ventilated indirect calorimetry (23). The room was ventilated with fresh air at a rate of 70 to 80 l/min. Flow was measured using electronically modified dry gasmeters (G6, gasmeterfabriek Schlumberger). The concentrations of O_2_ and CO_2_ were measured with dual pairs of infrared CO_2_ analyzers (ABB/Hartman & Braun Uras) and paramagnetic O_2_ analyzers (Servomex 4100 and ABB/Hartman & Braun Magnos) (23). During each 15-minute period, 6 samples of outgoing air, 1 sample of fresh air, zero gas, and calibration gas were measured. The gas samples to be measured were selected by a computer that also stored and processed the data (23). PA was continuously measured by an ActiSleep + (ActiGraph LLC) accelerometer worn on the hip. Participants had fixed bed times between 11:30 pm and 7:30 am. In the daytime, they were not allowed to sleep or to perform exercise. Meals were offered at stated times (breakfast: 9:00 am, lunch: 1 pm, dinner: 5:45 pm), and individuals were instructed to finish these within 30 minutes.

### Dietary intervention

Participants received either an MP diet (MP: 15:55:30 from En% protein:carbohydrate:fat) or an HP diet (HP: 25:45:30 En% from protein:carbohydrate:fat) corresponding with their dietary intervention instructions during the PREVIEW study (4). The basis of the meals was the same between groups, combined with either carbohydrate- or protein-rich food items to keep menus as comparable as possible. The diets consisted of commercially available food items and were provided individually in energy balance. Individual daily energy requirements were calculated as the basal metabolic rate using fat-free mass (FFM) and fat mass (24) multiplied by a PA level of 1.35 (25). Daily energy intake was divided over 3 meals, with breakfast containing 20%, and lunch and dinner 40%. During all measurements in the respiration chamber, the meals within each condition had the same macronutrient composition. Water consumption was allowed ad libitum between the meals; no other foods or beverages were available.

### Energy expenditure and respiratory quotient

Total energy expenditure (TEE) was determined during the 48-hour stay in the respiration chamber. O_2_ consumption and CO_2_ production were used to calculate TEE according to the Weir formula (26). Energy balance was calculated by subtracting TEE from energy intake. Respiratory quotient (RQ) was calculated by dividing CO_2_ production by O_2_ consumption as a measure of substrate oxidation.

### Anthropometric measurements

Body weight and composition were determined with individuals in the fasted state before entering the respiration chamber. Body weight was measured using a calibrated scale (Life Measurement Corporation, Inc). Body composition was determined based on body density measured via air-displacement plethysmography with the BodPod system (BOD POD, Life Measurement Inc) using Siri’s equation for body density (27). Height was measured using a wall-mounted stadiometer to the nearest 0.1 cm (Seca, model 222).

### Metabolic parameters

On the first and last day of the respiration chamber experiment, fasting blood samples were taken from an antecubital vein by venipuncture for the analysis of fasted glucose and insulin. In the morning of the second day, a venflon catheter (Becton, Dickinson and Company) was placed in the antecubital vein to collect fasted, preprandial, and postprandial blood for the analysis of endocannabinoid and related compounds. Blood was drawn through an airtight lock in the door, which had a plastic “sleeve” attached on the participants’ side of the lock. Participants were instructed to put their arm in the sleeve and to close it tightly around before the lock was opened. This enabled us to draw blood without affecting pressure inside the chamber. Blood samples were drawn directly before and 60 minutes after all 3 meals, with 1 sample more for the endocannabinoids and related compounds analyses 120 minutes after dinner. Samples were immediately stored on ice, centrifuged for 10 minutes at 1500 *g* at 4 °C, immediately distributed in aliquots, and stored at –80 °C until analysis at the end of the study, enabling all samples from one participant to be in the same analytical run.

### Glucose

Plasma for colorimetric glucose analysis (Roche Diagnostic Systems) was collected in sodium fluoride tubes (Becton, Dickinson and Company).

### Insulin

Serum for insulin analysis was collected in serum separator tubes (Becton, Dickinson and Company). Serum samples were kept at room temperature for 30 minutes to allow clotting before centrifugation (10 minutes at 1500 *g* at 4 °C). Samples were used to analyze fasting and postprandial insulin concentrations with a human insulin-specific radioimmunoassay (Linco Research). Insulin sensitivity was estimated by calculating the HOMA-IR (28).

### Endocannabinoids and endocannabinoid-related compounds

For endocannabinoid analysis, ice-chilled EDTA tubes (Becton, Dickinson and Company) were used. Samples were distributed in aliquots in prepared storage cups with 1% phenylmethanesulfonyl fluoride (PMSF) solution (10 mg PMSF in 1 mL methanol) and 5% 1 N HCL at final concentration. The set of different biochemical steps for the extraction, purification, and quantification of AEA, PEA, OEA, and 2-AG from plasma have been described previously (12, 14, 20, 29, 30). Subsequently, plasma samples were subjected to isotope-dilution liquid chromatography–chemical ionization-tandem mass spectrometric analysis. Mass spectral analyses were performed on a TSQ Quantum Access triple-quadrupole instrument (Thermo-Finnigan) equipped with an atmospheric pressure chemical ionization source and operating in positive ion mode (12). To evaluate between-run precision and reproducibility, quality control samples were prepared by directly supplementing a plasma pool control with endocannabinoids and run for each batch of samples analyzed. The amounts of AEA, PEA, OEA, and 2-AG are expressed as picomoles per milliliter of plasma.

Pregnenolone was extracted by a simple solid-phase extraction method using reverse-phase C18 columns from EDTA plasma, as described by Vallée et al (19). The steroid fraction was eluted with methanol (2 mL) into screw-cap test tubes and evaporated to dryness at 50 °C under a nitrogen stream to prepare for derivatization. Dried methanol extracts of plasma and standards were then derivatized by a 2-step procedure. The formation of pentafluorobenzyloxime for negative chemical ionization detection was followed by trimethylsilyl ether formation for adequate sensitivity. Samples were then subjected to isotope-dilution gas chromatography–chemical ionization-tandem mass spectrometric analysis. The derivatized samples were injected (1 μL) directly into a GCMSMS XLS Ultra Thermo mass spectrometer (Thermo-Finnigan) via an AS3000 II autosampler. The instrument was employed in negative ion chemical ionization mode, and a 15 m Rtx-5Sil MS W/Integra Guard capillary column (Restek) with a 0.25-mm inside diameter and 0.1-μm film thickness was employed for analyte resolution. To evaluate between-run precision and reproducibility, quality control samples were prepared by directly supplementing a plasma pool control with steroids and run for each batch of samples analyzed. The amount of pregnenolone was then expressed as nanogram per milliliter of plasma.

### Urinary nitrogen

During both days in the chamber, 24-hour urine was collected for the analysis of urinary nitrogen excretion as an estimation of protein metabolization (Vario Max, CN-analyzer, Elementar Analysesysteme GmbH). Urine bottles were prepared with 10 mL hydrochloric acid (4 mmol/L) to prevent nitrogen degradation. Nitrogen levels were then multiplied by 6.25 to calculate the daily protein intake (g/day).

### Statistical analysis

All statistical tests were performed using SPSS for Macintosh (version 25; SPSS Inc). Data are presented as means ± SD. Significance was defined as *P* less than .05. Normality of the parameters was assessed using the Shapiro-Wilk test, and outliers were detected with the use of box plots in SPSS. In the whole group, BMI, activity energy expenditure, and areas under the curve (AUCs) of activity-induced energy expenditure (AEA), AG, and OEA were not normally distributed. In the HP group, protein oxidation, fat oxidation, BMI, FFM, body-fat percentage, TEE, and activity energy expenditure were not normally distributed. In the MP group, AUCs of AEA, AG PEA, and OEA also were not normally distributed. For these reasons, nonparametric tests were used for the analyses. Differences between both groups were calculated using Mann-Whitney U tests. Associations between endocannabinoid concentrations were assessed using mixed linear model analysis. Changes in endocannabinoids during the day were assessed with repeated-measures analysis of variance followed by least significant difference posttest. Repeated-measures analysis of covariance (ANCOVA) and Spearman correlation analysis were used to assess possible relations between endocannabinoids and anthropometric variables, energy balance, substrate oxidation, and insulin resistance.

## Results

Anthropometric variables, HOMA-IR, energy intake, and energy expenditure did not differ significantly between the HP and MP (31). Energy balance was significantly lower in the HP group compared to MP (*P* = .015); RQ also was significantly lower in the HP group (31) (Table 1). With respect to the endocannabinoids and related compounds, AEA concentrations were positively associated with 2-AG (estimate 0.008; CI: 0.004-0.011; *P* < .001), PEA (estimate 0.060; CI: 0.050-0.069; *P* < .001), and OEA (estimate 0.072; CI: 0.061-0.083; *P* < .001) concentrations, assessed with mixed linear model analysis (Table 1). Likewise, PEA concentrations were positively associated with OEA (estimate 0.592; CI: 0.495-0.689; *P* < .001) and 2-AG (estimate 0.269; CI: 0.081-0.457; *P* < .01) and OEA concentrations were positively associated with 2-AG (estimate 0.072; CI: 0.061-0.083; *P* < .05). PREG concentrations were not associated with the other endocannabinoid concentrations.

**Table 1.** Anthropometric variables in medium-protein and high-protein groups at baseline, and when entering the respiration chamber

	Baseline			Respiration chamber		
	MP (n = 18)	HP (n = 20)	*P*	MP (n = 18)	HP (n = 20)	*P*
Sex, F/M	9/9	13/7				
Age, y	61.5 ± 5.7	59.4 ± 67.7	.443			
BMI, kg/m^2^	31.2 ± 4.1	30.0 ± 3.4	.553	29.0 ± 3.8	28.9 ± 4.0	.942
Fat-free mass, kg	54.7 ± 11.9	52.7 ± 11.9	.654	52.5 ± 10.9	50.8 ± 11.3	.553
Fat mass, kg	39.3 ± 9.0	36.5 ± 6.8	.346	33.9 ± 7.7	34.8 ± 8.8	.740
Body fat, %	41.9 ± 6.8	41.2 ± 6.3	.613	39.3 ± 7.4	40.7 ± 7.7	.593
HOMA-IR	3.4 ± 2.0	2.9 ± 1.3	.460	3.8 ± 1.8	3.62 ± 1.34	.794
EI, MJ/d				9.4 ± 1.7	9.3 ± 1.6	.573
TEE, MJ/d				9.2 ± 1.6	9.8 ± 1.7	.534
EB, MJ/d				0.2 ± 0.9	–0.5 ± 0.9	.015
RQ				0.84 ± 0.02	0.82 ± 0.02	.004

Data are presented as mean ± SD. Differences between groups were assessed by means of Mann-Whitney U tests, and the corresponding *P* values are shown in the table. Different changes from baseline to respiration chamber between groups were assessed by means of analysis of covariance, with baseline values as covariate; there were no significantly different changes between the groups.

Abbreviations: BMI, body mass index; EB, energy balance; EI, energy intake; F, female; HOMA-IR, homeostatic model assessment of insulin resistance; HP, high protein; M, male; MP, moderate protein; ; RQ, respiratory quotient; TEE, total energy expenditure.

**Figure 1.**
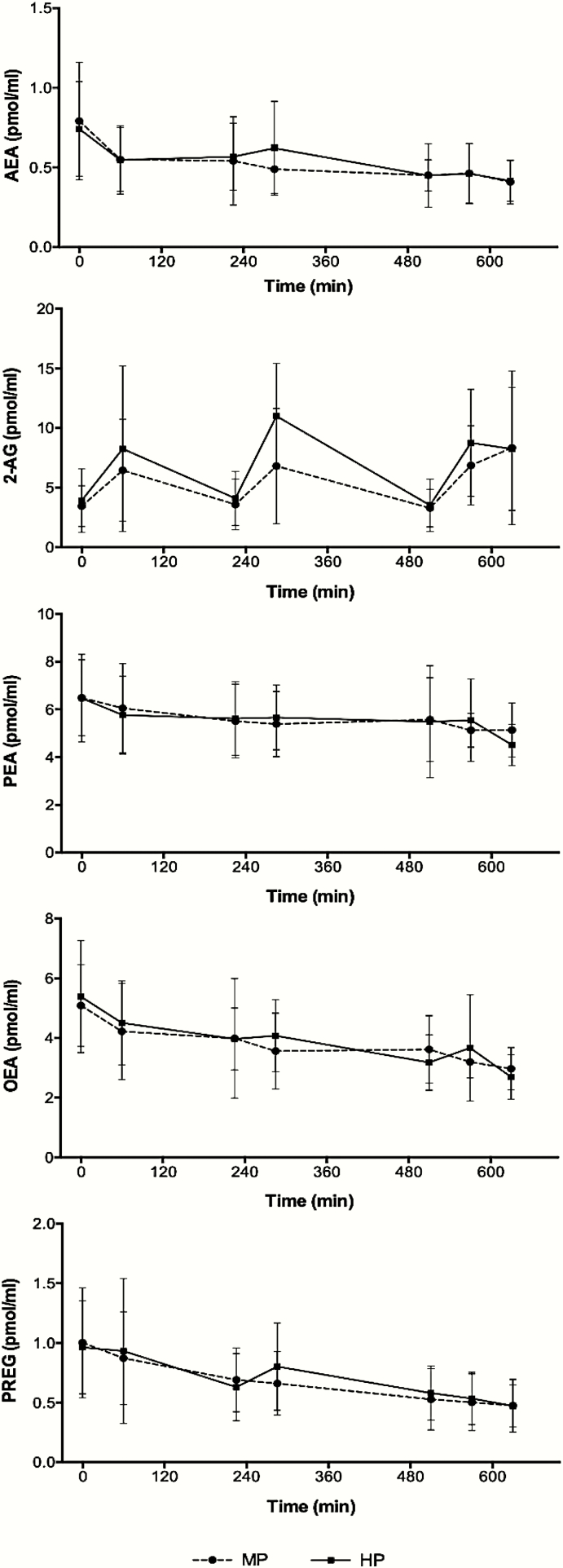
Endocannabinoid concentrations during the day in the medium-protein (MP) and high-protein (HP) groups. 2-AG indicates 2-arachidonoylglycerol; AEA, anandamide; OEA, oleoylethanolamide; PEA, palmitoylethanolamide; PREG, pregnenolone.

The AUC, from before breakfast to 120 minutes after the start of dinner, of the plasma concentrations of AEA, OEA, PEA, and PREG were not significantly different between the 2 groups. In contrast, the AUC of the plasma concentrations of 2-AG was significantly higher in the HP compared to the MP group (4351 ± 1616 vs 3368 ± 1552, F = 4.67, *P* < .05) (21). There were no significant group*time interactions between the groups, assessed with repeated-measures analysis of variance (Fig. 1). Therefore, the 2 groups have been combined for further analyses.

In the whole group, body-fat percentage was a significant predictor for AEA (*P* < .01; η _p_^2^ = 0.19), 2-AG (*P* < .05; η _p_^2^ = 0.13), PEA (*P* < .05; η _p_^2^ = 0.14), and OEA (*P* < .05; η _p_^2^ = 0.16) concentrations, assessed with repeated-measured ANCOVA. These results were also reflected by significant positive associations between the AUC of the endocannabinoid concentrations of AEA (r = 0.43), 2-AG (r = 0.37), PEA (r = 0.34), and OEA (r = 0.40) and body-fat percentage (*P* < .05), indicating that those individuals with a higher body-fat percentage had higher levels of these endocannabinoids and related compounds. In contrast, the AUC of PREG concentrations was not related to body-fat percentage. We also assessed associations between TEE and endocannabinoid concentrations using repeated-measures ANCOVA, correcting for FFM. TEE was a positive contributor to AEA (*P* < .05; η _p_^2^ = 0.17) and OEA (*P* < .05; η _p_^2^ = 0.12) concentrations. No relationships appeared with energy balance or HOMA-IR for any of the endocannabinoid concentrations and related compounds.

**Figure 2.**
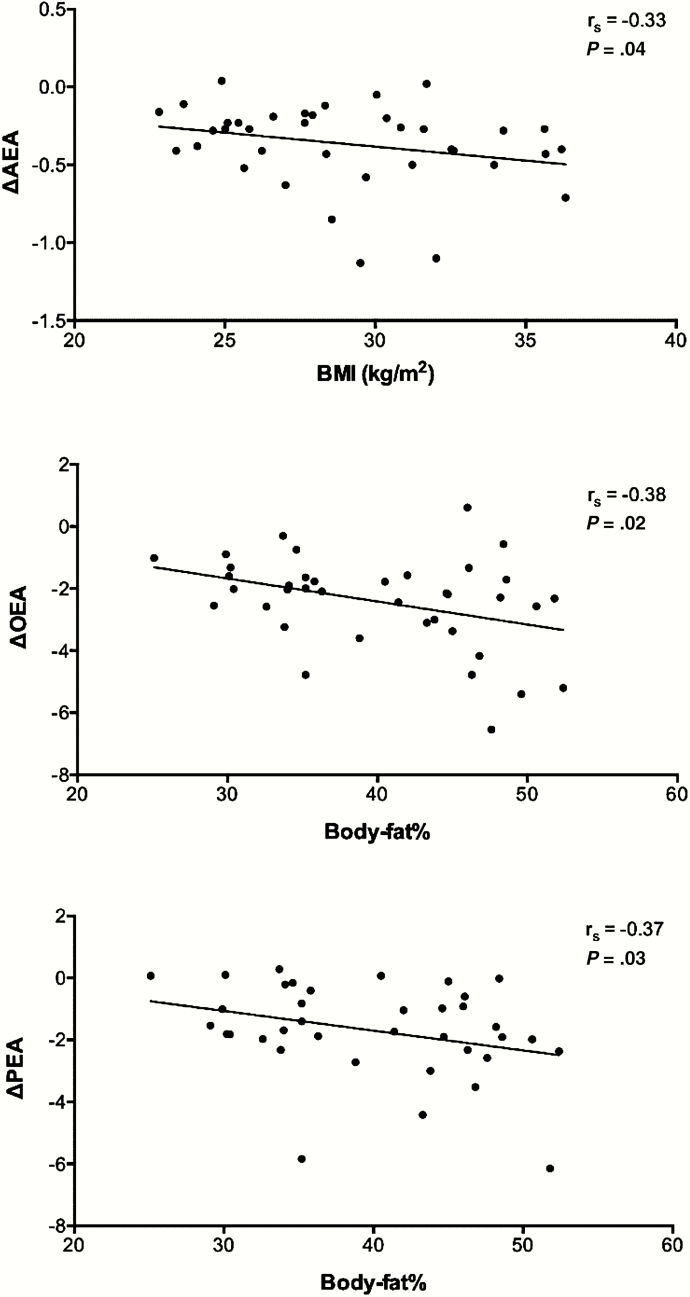
Associations of change in endocannabinoid concentration during the day and body mass index (BMI) (kg/m^2^) and body fat percentage assessed with Spearman correlation analysis. AEA indicates anandamide; OEA, oleoylethanolamide; PEA, palmitoylethanolamide.

**Figure 3.**
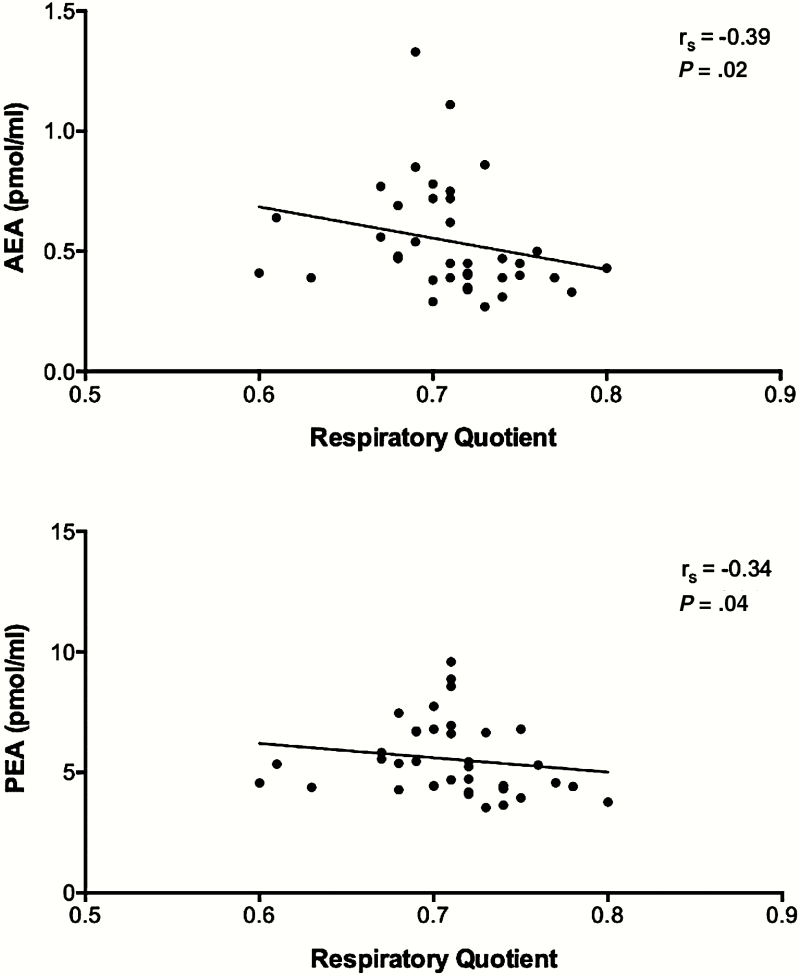
Associations of endocannabinoid concentration and respiratory quotient before lunch assessed with Spearman correlation analysis. AEA indicates anandamide; PEA, palmitoylethanolamide.

Interestingly, in the whole group the plasma concentrations of AEA, PEA, OEA, and PREG were the highest in the fasted state and decreased significantly during the day (Table 2), without showing any clear meal-associated pattern. The change in these concentrations from fasting to 120 minutes after dinner was inversely related to BMI (AEA: r = –0.33) or body-fat percentage (PEA: –0.37; OEA: –0.38) (Fig. 2); changes in PREG concentrations were not related to anthropometric values. Moreover, the RQ before lunch appeared to be the minimum RQ value over 24 hours, namely RQ = 0.71 ± 0.04. This RQ value was inversely associated with concentrations of AEA (r = –0.39; *P* = .016) and PEA (r = –0.34; *P* = .04) before lunch (Fig. 3).

**Table 2.** Concentrations of AEA (pmols/mL), 2-AG (pmols/ml), PEA (pmols/mL), OEA (pmols/mL), and PREG (ng/mL) before each meal and 60 minutes later, and 120 minutes after starting dinner

	B0	B60	L0	L60	D0	D60	D120
AEA^*a*^	0.77 + 0.33^*c*,*d*,*e*,*f*,*g*,*h*^	0.56 + 0.20^*b*,*f*,*g*,*h*^	0.56 + 0.24^*b*,*f*,*g*,*h*^	0.56 + 0.24^b,*f*,*g*,*h*^	0.45 + 0.16^*b*,*c*,*d*,*e*^	0.47 + 0.19^*b*,*c*,*d*,*e*,*h*^	0.41 + 0.13^*b*,*c*,*d*,*e*,*g*^
2-AG^*a*^	3.65 + 2.25^*c*,*e*,*g*,*h*^	7.51 + 5.87^*b*,*c*,*f*^	3.88 + 2.22^*c*,*e*,*g*,*h*^	9.11 + 5.07^*b*,*d*,*e*^	3.42 + 1.91^*c*,*e*,*f*,*g*^	7.91 + 4.08^*b*,*d*,*f*^	8.31 + 5.80^*b*,*d*,*f*^
PEA^*a*^	6.47 + 1.72^*d*,*e*,*f*,*g*,*h*^	5.89 + 1.76^*h*^	5.62 + 1.54^*b*,*h*^	5.55 + 1.38^*b*,*h*^	5.57 + 2.08^*b*,*h*^	5.38 + 1.37^*b*,*h*^	4.74 + 1.00^*b*,*c*,*d*,*e*,*f*,*g*^
OEA^*a*^	5.23 + 1.66^*c*,*d*,*e*,*f*,*g*,*h*^	4.40 + 1.50^*b*,*e*,*f*,*g*,*h*^	4.00 + 1.56^*b*,*f*,*h*^	3.86 + 1.26^*b*,*c*,*h*^	3.38 + 1.04^*b*,*c*,*d*,*h*^	3.46 + 1.36^*b*,*c*,*h*^	2.80 + 0.73^*b*,*c*,*d*,*e*,*f*,*g*^
PREG^*a*^	0.98 + 0.42^*d*,*e*,*f*,*g*,*h*^	0.90 + 0.52^*d*,*e*,*f*,*g*,*h*^	0.66 + 0.28^*b*,*c*,*d*,*f*,*g*,*h*^	0.74 + 0.33^*b*,*c*,*f*,*g*,*h*^	0.56 + 0.24^*b*,*c*,*d*,*e*,*g*,*h*^	0.51 + 0.23^*b*,*c*,*d*,*e*,*f*,*h*^	0.46 + 0.18^*b*,*c*,*d*,*e*,*f*,*g*^

B0, L0, D0: before breakfast, lunch, dinner; B60, L60, D60, D120: 60, respectively, 120 minutes after the start of breakfast, lunch, and dinner.

Abbreviations: 2-AG, 2-arachidonoylglycerol; AEA, arachidonoylethanolamide; OEA, oleoylethanolamide; PEA, palmitoyethanolamide; PREG, pregnenolone.

^*a*^Significant time interaction *P* less than .001 assessed with repeated-measures analysis of variance.

^*b*^Significantly different from B0.

^*c*^Significantly different from B60.

^*d*^Significantly different from L0.

^*e*^Significantly different from L60.

*f*Significantly different from D0.

^*g*^Significantly different from D60

^*h*^Significantly different from D120

Lastly, we have assessed the relationship between change in BMI or body-fat percentage prior to the respiration chamber measurements and endocannabinoid concentrations during the day. Changes in BMI (%) positively predicted 2-AG concentrations (η _p_^2^ = 0.142 *P* < 0.05) but no other endocannabinoid concentrations.

Furthermore, there were no associations between body-fat percentage and endocannabinoid concentrations or between changes in BMI percentage or body-fat percentage and changes in endocannabinoid concentrations during the day.

## Discussion

The present 48-hour respiration chamber study, during which PREVIEW-participants in the postobese phase were individually fed in energy balance, and in controlled macronutrient proportions, offered an excellent opportunity to investigate the concentrations of endocannabinoids and related compounds throughout the day, the changes thereof, and the possible interaction with energy balance. Although we did not find associations between the endocannabinoid concentrations and energy balance or adiposity, reductions in the concentrations of AEA, OEA, PEA, and PREG during the day were inversely related to BMI and body-fat percentage.

In the whole group of participants, we found statistically significant decreases in concentrations from fasting to 120 minutes after dinner in AEA, OEA, PEA, and PREG. This may suggest that these endocannabinoids respond to a gradual energy supply to meet energy balance during the day. This is also in line with the finding that AEA and OEA were positively associated, corrected for FFM. Participants with a higher energy turnover presented with higher concentrations of AEA and OEA, which may again be an effort to meet energy balance during the day. This was further underscored by the lowest RQ during the day, before lunch, being inversely associated with concentrations of AEA and PEA before lunch. The significant decrease of AEA, OEA, PEA, and PREG may point toward a circadian rhythm in the secretion of these compounds, as was suggested previously (32). OEA and PEA changed in the same direction as AEA, which can be explained by the fact that these compounds, although physiologically playing different roles, are synthesized along the same intracellular pathways (9). Again, this may be due to the controlled study design of feeding participants in energy balance, which does not imply strong feelings of craving or fullness.

Moreover, the changes in concentrations of AEA, OEA, and PEA were inversely related to BMI (AEA) and body-fat percentage (PEA, OEA). This may indicate that those with a lower BMI or fat mass presented with a stronger energy-balance regulation compared to those with a higher BMI or fat mass, who could easily live on their reserves for a longer period of time. The lack of a relationship of pregnenolone concentrations with anthropometric measurements underscores the different functionality of pregnenolone inhibiting the CB1R (19, 20), which likely takes place only on important and sustained activation of CB1R.

With respect to the effect of diets with different protein and carbohydrate compositions on endocannabinoid concentrations and interactions with energy balance, we observed no statistically significant differences in AEA, OEA, PEA, and PREG concentrations between the 2 diets used in the study. Only the AUC of 2-AG was statistically significantly different between the diets in that it was higher in the HP diet group compared to the MP diet group (21). Moreover, the concentrations of AEA, 2-AG, OEA, PEA, and PREG were not associated with energy balance, energy expenditure, or insulin sensitivity, independently of diet group. This is in contrast with previous studies that underscore the hypothesis of AEA being an orexigenic factor showing a pattern of changes in circulating AEA concentrations around meals (29, 33, 34). AEA concentrations have been observed to be increased before meals in individuals with normal weight and with obesity (29) and to be decreased after food consumption (11, 33, 34), independently of the hedonic value of the meal in participants of normal weight (35). Furthermore, it was observed that the endocannabinoids may affect energy balance in that AEA and 2-AG would promote energy intake and might be inhibited by PREG, whereas OEA and PEA would promote satiety or energy expenditure (17-20, 36). Moreover, AEA has been suggested to affect energy expenditure by mediating the effect of skeletal muscle sphingomyelins (37). Finally, associations of peripheral endocannabinoids with energy expenditure in Native Americans have been reported (38). That we did not observe any of those effects may be due to the controlled design of the study, with participants being fed to their individual energy balance, and having to finish the food that was offered to them, without the possibility of hedonic eating or overeating (33, 34). To our knowledge this is the first study to investigate the relationship between endocannabinoid excursion during the day, adiposity, and energy metabolism in a controlled study design during the postobese phase, while fed in energy balance.

Interestingly, we found a positive relation between the degree of weight loss prior to the respiration chamber measurements and 2-AG concentrations during the respiration chamber, with higher weight loss being associated with lower 2-AG concentrations, possibly caused by a reduction in ghrelin and hunger (18). Moreover, in the present conditions we did not observe any associations with insulin sensitivity, which have been reported before (15), although our participants still presented with the characteristics of prediabetes. Being previously obese, participants were in a postobese state on average, when being assessed in the respiration chamber. Although previous literature has shown links between circulating endocannabinoids and metabolic parameters in obese individuals, it is unclear how these relationships hold after people have lost weight. This relationship may depend on the timing of the measurements, for example, during weight loss or short-term after weight loss, when in fact the effect during negative energy balance is measured, or long-term after weight loss when energy balance has been established again. Studies investigating changes in endocannabinoid concentrations after bariatric surgery–induced weight loss found an increase in AEA but not 2-AG levels 2 months after surgery (39) and a reduction in levels of 2-AG and AEA at 12 months after surgery (40). A 16-week very low-calorie diet led to a reduction in AEA levels, but not 2-AG levels (41) and a 1-year lifestyle program including healthy eating and PA led to a reduction both in 2-AG and AEA (42). Given the rather substantial weight change after surgery, our study adds to the body of literature by assessing postobese individuals while being fed in energy balance.

We need to point out a limitation regarding the design of the current study. We would have preferred to include the respiration chamber and endocannabinoid measurements at the start of the PREVIEW study as well. However, this was not feasible without putting too much strain on the participants. Furthermore, it has to be considered that the relationships between endocannabinoid concentrations and body-fat percentage and RQ that were reported in the present study would not survive correction for multiple comparisons (eg, Bonferroni correction). Therefore, the findings of the present study need to be confirmed in a larger group of participants. Even though meals were obtained from general supermarkets and were provided as close as possible to free living conditions, they may have differed slightly from food consumed in the home condition of the participants. Therefore, we cannot rule out that interactions between the diet group in the respiration chamber and in free-living conditions may have masked diet-specific effects. However, a possible interaction does not affect the main conclusions.

Taken together, we did not observe differences in the concentrations of endocannabinoids and related compounds due to differences in macronutrient compositions, except for 2-AG, which we interpreted as possibly related to a higher satiety. We did observe a significant decrease of AEA, OEA, and PEA concentrations during the day, probably indicating the gradual energy supply over the day. This decrease in endocannabinoid concentrations was inversely related to BMI or body-fat percentage, suggesting a stronger energy-balance regulation in those with a lower adiposity. In conclusion, in energy balance, only the endocannabinoid 2-AG changed in relation to protein level in the diet, whereas the endocannabinoid AEA and endocannabinoid-related compounds OEA and PEA reflected gradual energy intake matching energy expenditure during the day.

## Abbreviations

2-AG: 2-arachidonoylglycerol
AEA: anandamide
BMI: body mass index
CBRs: endocannabinoid type receptor 1 and 2
ECS: endocannabinoid system
HP: high protein
MP: medium protein
OEA: oleoylethanolamide
PA: physical activity
PEA: palmitoyethanolamide
PREG: pregnenolone
TEE: total energy expenditure
T2D: type 2 diabetes

## Additional Information

***Disclosure Summary:*** The authors have nothing to disclose.

***Data Availability:*** The datasets generated during and/or analyzed during the current study are not publicly available but are available from the corresponding author on reasonable request.
